# Individualized Dosing of Therapeutic Monoclonal Antibodies—a Changing Treatment Paradigm?

**DOI:** 10.1208/s12248-018-0257-y

**Published:** 2018-09-05

**Authors:** Anne S. Strik, Yow-Ming C. Wang, Laura E. Ruff, William Yashar, Bradley T. Messmer, Diane R. Mould

**Affiliations:** 1 Academic Medical Center Division of Gastroenterology, Amsterdam, Netherlands.; 2 Therapeutic Biologics Program, Office of Clinical Pharmacology, Office of Translational Sciences, Center for Drug Evaluation Research, Food and Drug Administration, Silver Spring, Maryland, USA.; 3 Abreos Biosciences, San Diego, California, USA.; 4 Projections Research Inc., 535 Springview Lane, Phoenixville, Pennsylvania 19460, USA.

**Keywords:** dashboard, inflammatory bowel disease, monoclonal antibodies, point of care, therapeutic drug monitoring

## Abstract

The introduction of monoclonal antibodies (mAbs) to the treatment of inflammatory bowel disease (IBD) was an important medical milestone. MAbs have been demonstrated as safe and efficacious treatments of IBD. However, a large percentage of patients either fail to respond initially or lose response to therapy after a period of treatment. Although there are factors associated with poor treatment outcomes in IBD, one cause for treatment failure may be low mAb exposure. Consequently, gastroenterologists have begun using therapeutic drug monitoring (TDM) to guide dose adjustment. However, while beneficial, TDM does not provide sufficient information to effectively adjust doses. The pharmacokinetics (PK) and pharmacodynamics (PD) of mAbs are complex, with numerous factors impacting on mAb PK and PD. The concept of dashboard-guided dosing based on Bayesian PK models allows physicians to combine TDM with factors influencing mAb PK to individualize therapy more effectively. One issue with TDM has been the slow turnaround of assay results, either necessitating an additional clinic visit for a sample or reacting to TDM results at a subsequent, rather than the current, dose. New point-of-care (POC) assays for mAbs are being developed that would potentially allow physicians to determine drug concentration quickly. However, work remains to understand how to determine what target exposure is needed for an individual patient, and whether the combination of POC assays and dashboards presents a safe approach with substantial outcome benefit over the current standard of care.

## INTRODUCTION TO INFLAMMATORY BOWEL DISEASE

Inflammatory bowel disease (IBD) includes two types of auto-immune disorders that cause prolonged inflammation of the digestive tract: ulcerative colitis (UC) and Crohn’s disease (CD). One difference between these two types of IBD is the location of inflammation. In CD, inflammation can occur throughout the entire gastrointestinal tract, from mouth to anus. In UC, the colon and rectum are primarily affected. Although CD and UC are different diseases, differentiation based on the clinical picture can be difficult. Both UC and CD may present with abdominal pain, diarrhea, rectal blood loss, weight loss, vomiting, or signs of anemia. Diagnosis is usually made based on endoscopic evaluation and biopsies taken for histopathological assessment. Not only are the locations of these two diseases different, associated complications also differ. CD can be characterized by late complications such as a stenosing and/or penetrating phenotype which can result in fibrostenotic strictures, (perianal) fistula, or abscesses; UC does not have late complications. Although the exact etiology is unknown, IBD is likely caused by a combination of genetic and environmental factors leading to an immunological response and intestinal inflammation (1,2). Historically, the goal of IBD treatment was to relieve symptoms; it has now shifted to the achievement of mucosal healing.

## INFLAMMATORY BOWEL DISEASE TREATMENT OPTIONS

In 1998, approval of the first monoclonal antibody (mAb), infliximab (IFX), for CD, provided an exciting new treatment option. Since then, multiple mAbs with diverse mechanisms of action have been marketed for IBD. Currently available drugs for treatment of IBD include chemical agents such as steroids, immunomodulators, Janus kinase (JAK) inhibitors, and mAbs such as the anti-integrins, anti-interleukin-12/23p40, and anti-TNF agents (3,4). Clinical development programs of IBD treatments generally follow a stepwise approach, beginning with initial evaluations of safety and tolerability, followed by evaluations of dose–/exposure–response (E–R) in small clinical studies, then phase 3 clinical trials that provide evidence of clinical efficacy and safety in the target patient population. Currently, most evidence about clinical and endoscopic outcomes is derived from clinical trials with mAbs.

### Current Recommended Dose Regimen for Inflammatory Bowel Diseases

The treatment paradigm for CD and UC involves two regimens: (1) initial treatment period (induction) and (2) second regimen (maintenance), which are supported by evaluations during clinical development programs. Maintenance regimens are typically less intensive either in the dose or frequency than for induction (Table I). Knowledge about mAb drugs for IBD continues to grow beyond data that supported regulatory approval, including collective experience from clinical use and post-marketing clinical trials. The learning continuum has influenced the philosophy and practice in IBD treatment, including active discussions of individualized dosing for biological drugs.

### MAb Pharmacokinetic Variation in IBD

MAbs generally exhibit complex pharmacokinetic (PK) behavior, whereby disposition involves neonatal-Fc receptor (FcRn)-antibody binding, which can be influenced by FcRn genetic polymorphism. Recently, the VNTR2/3 genotype in the FcRn gene was shown to be associated with lower mAb exposure during induction in IBD patients (5,6). Intracellular catabolism, extracellular degradation (proteolysis), receptor-mediated and fluid-phase endocytosis of the mAb, target-mediated drug disposition, and *in vivo* post-translational modifications (PTM), such as deamidation (7–10) and nonenzymatic glycation (11), contribute to variability. High glycation is associated with diabetes, which was shown to result in a 28.7% increase in ustekinumab clearance (12); hyperglycemia was shown to increase IFX clearance up to 15% (13). Whether and how PTM differ across patients remain poorly understood; however, it may result in loss of mAb activity prior to clearance (14,15). In addition, mAbs with multimeric soluble targets can form immune complexes and be rapidly cleared via phagocytosis (7). Numerous other factors contribute to between-patient PK variability. These factors include patient demographics such as body size, serum albumin concentration, C-reactive protein (CRP), formation of anti-drug antibodies (ADAs), endogenous IgG concentration, expression/turnover of antibody target, mAb saturation of target, and drug–drug interactions. Many mAbs and biologics including certolizumab pegol (16–18), which is indicated only for CD, and golimumab (19,20), which is indicated only for UC, reported the same factors as influencing PK and showed that observed variability can negatively impact patient outcomes.

Pediatric patients present unique issues, given both growth and maturation effects (21) and the fact that diseases can present in a more aggressive form in pediatric patients than adults (22) suggesting potential etiologic differences from adult disease (23). Other disease-specific routes of clearance such as protein-losing enteropathy (24–27) can play an important role, as discussed later. Taken together, these factors contribute to high between-patient variability in mAb PK in IBD patients.

Two mAbs often used for treatment of IBD are IFX and adalimumab (ADL), administered intravenously and subcutaneously, respectively. Both anti-TNF agents have proven to be successful in inducing and maintaining clinical and endoscopic remission (28–32). However, a proportion of IBD patients do not respond to anti-TNF induction treatment (primary non-responders) or lose response during maintenance treatment (secondary non-responders) (33).

Individualized dosing for mAb drugs began to acquire clinical attention many years after biological drugs had made a substantial, positive impact on patients with CD, UC, and other disease conditions. Impetus to investigate dose individualization of mAb drugs stems from findings of IBD trials and clinical practice. Some important common findings are as follows:

1. Only a portion of patients achieve the predefined treatment response at the end of induction and continue into maintenance treatment.
2. Only a fraction of patients who receive maintenance treatment achieve treatment response.
3. Patients can lose treatment response after being in stable response for a period of time.

Incomplete response to induction treatment may be attributable to inadequate exposure or patient factors that preclude treatment response. Similarly, loss of response (LOR) to maintenance treatment may be due to inadequate exposure in general or secondary treatment failure due to immunogenicity against the mAb drug or non-immune-mediated processes.

Factors associated with primary non-response to anti-TNF agents are smoking, disease duration of more than two years, small bowel disease, and underdosing during the induction phase, presumably due to fecal loss. Brandse and colleagues demonstrated that IFX could be measured in feces of patients with acute severe colitis starting with IFX induction treatment (34). The highest fecal IFX concentrations were measured during the first few days after starting treatment. A possible explanation for this phenomenon is protein-losing enteropathy due to severe intestinal inflammation leading to a “leaky gut.” If IFX is lost via the feces, IFX exposure following regular induction of 5 mg/kg at week 0, 2, and 6 may be insufficient, necessitating higher anti-TNF doses or shorter dosing intervals. During IFX maintenance (5 mg/kg every eight weeks), serum drug levels between 3 and 7 μg/ml are considered therapeutic (35). However, during the IFX induction phase, higher serum drug levels are needed (e.g., ≥ 28 μg/ml during the first two weeks and ≥ 15 μg/ml in week 2 to 6) (36,37). In some patients, for example, patients with an increased clearance of the drug, higher IFX doses or shorter intervals may be needed to achieve these levels (38,39).

### Development of Anti-drug Antibodies

Development of immunogenicity against the mAb is a key concern because of the potential association with reduced systemic drug exposure and treatment effectiveness. ADA formation, i.e., immunogenicity, can lead to secondary LOR in IBD patients (40–42). While clinical concerns related to immunogenicity may be apparent today, it was not in the prescribing information associated with the initial approval of IFX in 1998 (43). The first mention of an association of immunogenicity with a reduced efficacy and increased IFX clearance appeared in the prescribing information 6 years later (44). To prevent LOR during maintenance treatment with mAb drugs, clinical research has led to new institutional recommendations for dosing in IBD treatment. These efforts are well-documented in publications (45–47), and encompass approved mAb drugs with diverse mechanisms of action.

ADA can form complexes with the mAb, either neutralizating biologic effect and/or accelerating clearance. Since immunogenicity is often accompanied by subtherapeutic serum drug levels, maintaining adequate mAb concentrations is crucial in the treatment with anti-TNF agents. Current strategies to reduce the immunogenicity include scheduled instead of episodic treatment with anti-TNF agents and combination treatment with an immunomodulator, such as methotrexate or a thiopurine. However, if ADA does occur in patients treated with anti-TNF monotherapy, addition of immunomodulators during treatment can be used to suppress ADA. Research in IBD patients showed that addition of immunomodulators to anti-TNF monotherapy in patients with LOR due to immunogenicity led to increased serum drug levels, accompanied by ADA decrease to undetectable levels (48–50). The exact mechanism behind this observation is unknown, but research in rheumatoid arthritis patients treated with methotrexate demonstrated downregulation of activating Fc-gamma receptors, which could be a direct effect of methotrexate on monocytes potentially associated with the disease-modifying effect of methotrexate (51).

### Maintaining Effective mAb Exposure—Introduction of Therapeutic Drug Monitoring

Potential subtherapeutic exposure is another key area of clinical investigations. Most IBD trials evaluate efficacy and safety at one or two dose regimens, which produce a range of systemic exposure as measured by the trough concentrations. In some trials, dose escalation based on clinical response may be an option; however, trial design often hampers robust evaluation of the benefit of dose escalation. Consequently, clinicians continue to investigate various dosing paradigms to benefit as many patients as possible with existing medicines, a quest universally applicable to chemical drugs and biological drugs. Literature reports have revealed potential reasons for IBD treatment failure as shown in Table II grouped by two factors, systemic drug concentration and immunogenicity findings. These findings form the basis to proposing alternative strategies for clinical management.

Research has indicated that dosing according to a “one size fits all” principle is no longer optimal in IBD treatment. The observation that not all patients respond the same way to a certain dosing regimen emphasizes the need for adequate, more personalized dosing using therapeutic drug monitoring (TDM). There is a shift toward individualized dosing in which TDM plays an important role and is increasingly utilized by gastroenterologists.

TDM is defined as measuring patient responses (e.g., drug concentrations, ADA, biomarkers, or clinical outcomes) and adjusting the therapy (e.g., the dose amount or frequency) accordingly, where ADA is relevant to mAbs for IBD treatment. This is particularly true for treatment with mAbs and thiopurines, with the former agents exhibiting highly variable clearance and the latter agents having a narrow therapeutic range. Patient factors influencing the PK of a drug should also be taken into account. For example, multiple analyses of IFX population PK data derived from IBD patients identified multiple factors influencing the clearance of IFX; the presence of ADA, low serum albumin, high serum CRP, and high body weight are the most important factors associated with increasing clearance of anti-TNF agents (38,39), and these same factors also influence clearance of other mAbs.

By incorporating these factors in a PK model and using Bayesian approaches, predictions can be made to identify dose intervals to maintain a certain serum concentration. Since most gastroenterologists are not used to working with model-based dosing, a tool for physicians to apply TDM in the most optimal way is preferred. A dashboard system is an example of such tool, increasingly used by physicians to individualize dosing of their patients (52,53). With a PK model, incorporated patient factors, and a Bayesian approach, the dashboard system utilizes the concentration data from the previous dose and calculates the exact dosage a patient should receive and the exact date this dosage should be given to maintain a certain drug concentration. To make this prediction as accurate as possible, frequent blood sampling throughout treatment is desirable, although not necessary (54,55). However, care should be taken to ensure that the underlying structural model in the dashboard is appropriate. Dubinsky *et al.* compared dashboard-driven dosing with actual administered dosing regimens in the standard-of-care setting (56). Serum drug level measurements were derived from pediatric IBD patients using IFX at week 14 and 54 after start of therapy. Dashboard-guided dosing after week 14 was compared to the actual administered IFX dose and frequency given with standard care. In the majority of the cases, the dashboard-recommended dose and/or interval changes instead of the regular dosing regimen. To investigate the clinical benefit of dashboard-driven dosing in IBD patients treated with mAbs, a prospective trial comparing the two dosing regimens should be performed.

### Important Drivers for Individualized Dosing

The success of individualized dosing depends on many important factors, e.g., a predefined clinical outcome, the desired systemic exposure (range) to achieve the predefined clinical outcome, patient factors driving PK exposure, patient factors driving the treatment response (including characteristics of patients who will not respond to treatment), immunogenicity, and enabling technical tools. The latter covers those for measuring patient factors, biomarkers, clinical outcomes, drug concentrations, and ADA, and for identifying patient factors through PK modeling, PD modeling, or integrated PKPD modeling.

Desired treatment outcomes are defined by clinicians and reflected in clinical trials as study endpoints, which may evolve over time with increasing knowledge of the disease and the advancement of technology. For example, clinical response, clinical remission, mucosal healing, and endoscopic remission are some of the endpoints used in IBD trials in addition to exploratory biomarkers (57,58). While an effective treatment would achieve the desired clinical outcomes in all treated patients, in reality, there are subgroups that do not reach the desired effect despite achieving high systemic exposures for reasons that are not well understood (59). Conversely, some patients may achieve the outcomes despite having relatively low systemic exposure (59). Pediatric patients present an acute challenge because of the scarcity of clinical data supporting drug usage and dosing in children, a need recognized by regulatory agencies (60) and owing to the desire to maintain comparable exposures in pediatric patients with adults based on the assumption of similar disease progression and similar exposure-response between adult and pediatric patients.

To move beyond empirical experimentation, proactive identification of patient factors that influence treatment response is very important as illustrated in Table II and is an area for further research. In contrast, patient factors driving PK are much better studied owing to data availability and application of PK modeling tools. The experience with use of dashboard systems (56) for IFX dose optimization shows the potential utility of patient intrinsic factors in PK-driven individualized dosing in IBD. Acute severe ulcerative colitis patients starting induction treatment with IFX and patients on IFX maintenance treatment losing response due to subtherapeutic drug levels will presumably benefit most from dosing based on serum drug measurements as these patients have very high mAb clearance (61). Notably, some mAb drugs are supplied with a fixed amount in prefilled syringes or autoinjectors for subcutaneous injection intended for self-administration, which may present a challenge to fine-tuning doses for individual patients.

#### Defining Desired Systemic Exposure (Range)

Although defining the desired systemic exposure is not among the preset objectives of confirmatory efficacy and safety trials, these trials generally report steady-state trough concentrations at the recommended dose in the intended patient population. Additional real-world data from post-marketing experience, such as institutional investigations, can contribute to the body of knowledge to inform the desired systemic exposure. However, uncertainties exist as to the suitability of using the observed steady-state concentrations as the target concentration for individualized dosing given that measures for desired outcome differ across various studies and may differ from that expected in current clinical practice. As shown in the review of IBD literature conducted by Papamichael *et al.* (58), therapeutic concentrations vary with the desired outcome or the predefined clinical endpoints as well as the timing of clinical outcome assessment with respect to the treatment duration. Work to identify appropriate concentrations for certolizumab pegol (16,17) and golimumab (19,20) has also been published.

Several recent retrospective investigations on proactive dose optimization (52,56) were published. Publications of institutional experience with IBD treatments have identified the respective target drug concentrations for reactive dose optimization. For clinically managing IBD patients each reporting institution naturally would rely on their own scientific findings. To facilitate a broader application, the American Gastroenterology Association published a paper with recommendations for reactive TDM (62). Although not all studies used the same bioanalytical method to measure drug concentrations, independent reviewers from the society have indicated that results from various assays appear to be generally in good agreement (62,63) although a retrospective study showed that some assays do not have good performance (54).

The importance of identifying appropriate drug exposure is highlighted by natalizumab, a humanized IgG4κ mAb initially approved in 2004 for multiple sclerosis (MS) and approved in 2008 for the treatment of CD. It was subsequently withdrawn from the market by its manufacturer after being linked with three cases of progressive multifocal leukoencephalopathy (PML). PML is an often-fatal opportunistic brain infection caused by the John Cunningham (JC) virus. Patients taking natalizumab are at risk of PML if they are JC virus antibody positive (~ 55% of all patients). It is hypothesized that high concentrations of natalizumab may affect immune cells’ ability to traffic to the brain and control the JC virus (64,65). In MS patients, excessive dosing of natalizumab has been linked to increased PML risk (66,67). In a recent study, extended-interval dosing (dosing every 5–12 weeks instead of the standard 4-week dosing) decreased PML risk by 94% (67). Therefore, PML risk can be mitigated by extended-interval dosing (but not changing the administered dose), which effectively decreases the concentration of natalizumab.

#### Defining the Effect of Immunogenicity

Immunogenicity was shown to negatively affect the systemic exposure and clinical outcomes; however, the role of ADA data in clinical management of IBD patients is not well defined. The proportion of patients who achieved desired clinical outcome is smaller in ADA-positive patients as compared to ADA-negative patients, and an ADA-positive finding has been associated with lower systemic drug concentration (68–71). However, it has not been feasible to quantify the effect of ADA at the subject level or at the population level, in part because the immunogenicity data have been reported in a dichotomous manner, i.e., ADA positive and ADA negative, as described above. In recent years, ADA data are increasingly reported with titer values representing the intensity of the immunogenicity response. Currently, available data generally suggest that higher ADA titers are associated with worse clinical measures, such as reductions in systemic drug exposures, which generally precede exposure reduction or LOR (72). However, identifying the ADA titer thresholds that are clinically relevant is challenging because the characteristics (e.g., binding affinity, with or without neutralizing capability, proportion of ADA mixture being neutralizing) and the kinetics of ADA (e.g., temporal change in intensity and characteristics) may vary in patients. Furthermore, the diversity of assays used for measuring ADA titer across reported studies (some developed by institutional laboratories, others by regional laboratory service providers) presents challenges to the integration of literature reported data. Although the pursuit of quantifying the effect of ADA on PK has resulted in limited success (73), the availability of ADA titer data has fueled a new wave of investigations. From these studies, we will learn more about the clinical utility of ADA titer data in dose individualization for IBD treatment.

It is worth noting that immune-mediated reduction in systemic exposures is one of several possible mechanisms for LOR (62). Other processes not mediated by immunogenicity, e.g., disease flare-up, also can be associated with a reduction in drug concentrations. Among patients who experienced immune-mediated reduction in systemic exposure, the timing of measurement and the extent of exposure reduction can vary, with some patients showing no measurable concentrations at all. The same variability in drug exposure reduction is seen with the impact of disease flare. This variability can make dose adjustments difficult to standardize in the case of either ADA or disease flares.

### TDM Utility

The use of TDM for IBD has several issues, including the influence of disease activity on PK and the wide range of target trough values in the literature (74). TDM utility for mAbs has been questioned, partly because of a lack of powered prospective studies using TDM-based dosing as compared to standard of care (75). A large prospective trial (69) showed TDM-based dosing maintained response although in all patients dosing was optimized using TDM during the initial phase of this study. Even though TDM-based dosing was not superior compared to clinically based dosing, significantly more patients in the clinically based dosing group needed an intervention to treat a relapse. Likewise, more undetectable trough levels and ADA were seen in this group. A small prospective study (TAILORIX) investigated only dose increases in maintenance therapy over 1 year, but not shortened dosing intervals, an important adjustment. The study design was not sufficient to demonstrate an advantage of TDM (76) but suggested no benefit. Both trials only included patients on IFX maintenance and had a follow-up of 1 year, which, given the annual rate of LOR for IFX [10–20% (77)], might be too short to show real benefit.

An important issue that should be addressed is the benefit from proactive TDM compared to reactive TDM. A recent review of TDM (80) suggested that the true value of TDM may be preemptive dose optimization to prevent LOR. A randomized controlled trial reported that preemptive TDM-based dose optimization decreased the risk of LOR compared to dosing of IFX based on symptoms and inflammatory biomarkers (35). Unfortunately, there is insufficient evidence to draw robust conclusions. However, in a retrospective analysis of IBD patients comparing proactive versus reactive monitoring of serum concentration of IFX, proactive monitoring was associated with better clinical outcomes, including greater drug durability, less need for IBD-related surgery or hospitalization, and lower risk of ADA or serious infusion reactions (81).

Most IBD treatment regimens contain a higher-intensity dosing for induction followed by a lower-intensity dosing for maintenance, indicating that achieving adequate serum drug levels during induction is as important as during maintenance. Studies have illustrated that trough concentrations during induction are higher than the trough concentrations during maintenance (79). However, investigations into whether patients who do not respond to conventional induction regimen of mAbs for IBD could benefit from dose individualization have not been conducted. The strategy for dose individualization or preemptive TDM during induction warrants further research. The anticipated challenges would include defining the biomarkers to use and their baseline values for guiding the induction dose regimen.

Another area of interest is the economic impact of TDM. The study of Steenholdt *et al.* showed substantially lower (34%) costs for those randomized to the TDM arm compared to IFX dose intensification to 5 mg/kg every 4 weeks (€ 6038 vs € 9178, *p* < 0.001) in IBD patients with secondary LOR (78). The economic superiority of TDM-based dosing was maintained throughout 1 year. The TAXIT study (35) also found a slight economic advantage to TDM-guided dosing. However, evaluating treatment costs is complex, for example, the costs of additional drugs (steroids, immunosuppressants), laboratory tests, hospitalization, and surgery should be considered.

For both ustekinumab and vedolizumab, there are currently insufficient data to support routine use of TDM although data from the confirmatory efficacy and safety studies may provide insights into the desired trough concentrations in IBD patients (82–85). Further studies to elucidate the role of TDM in the treatment of these agents are needed. TDM utility for mAbs has also been questioned due to slow assay turnaround, analytical deficiencies, assay differences, and difficulties with interpreting results (86). Moreover, immunogenicity response may differ across treated patients. As such, an overall TDM strategy that considers patients’ immune responses in addition to drug concentration may be beneficial albeit it would differ from traditional TDM for small molecule drugs which is largely based on drug concentrations and aims at reducing the likelihood of adverse events.

## TDM ASSAYS FOR PRECISION DOSING OF BIOLOGIC DRUGS

### TDM Assays for Biologics

In IBD, multiple diagnostic companies offer TDM tests for IFX, due to the breadth of published literature identifying specific drug concentration ranges for optimal patient outcomes (87–89). MAb TDM test results are then combined with other monitoring tests, such as ADA, serum albumin, and CRP measurements, to adjust treatment to manage disease flares and treatment failure (90).

There are currently no FDA-cleared TDM tests for any mAb. Marketed mAb TDM tests in the USA are all laboratory-developed tests (LDTs), and cover only a small portion of the approved biologics (mostly those indicated to treat IBD). Most TDM LDTs utilize a sandwich or bridging enzyme-linked immunosorbent assay (ELISA). In this format, either a recombinant antigen or an anti-idiotype antibody is used as a capture reagent. A recombinant antigen, anti-idiotype antibody, or anti-isotype antibody is used as a labeled detection reagent. While tests for the same therapeutic mAbs are available from several different diagnostic companies, there is a lack of standardization for the different assays (91). Cell-based assays, mass spectrometry, and mobility shift assays for mAb TDM also exist. In cell-based assays, the target molecule is expressed on a cell line by transfection, and flow cytometry is used to measure binding of the desired mAb. This method has been used for PK analysis of alemtuzumab (anti-CD52) (92). Cell-based assays, however, are labor intensive, requiring specially trained personnel and expensive equipment. Liquid chromatography–mass spectrometry (LC–MS), monoclonal Ig rapid accurate measurement (miRAMM), and Orbitrap are different mass spectrometry techniques used to quantitate mAb concentrations. These methods detect either tryptic peptides or intact light chains to quantitate mAbs. In the former, digested mAbs must have peptide fragments that are unique to the drug and not present in the polyclonal antibody background of serum (~ 10 mg/ml); this poses a problem for some fully human mAbs, such as ADL. In the latter, the reliance on intact light chains for detection and quantitation allows detection of fully human antibodies; however, the assay may not be sensitive enough for low-concentration measurements, such as trough concentration for some drugs (93). Currently, mass spectrometry-based TDM LDTs for IFX and eculizumab are available (94,95). Finally, homogenous mobility shift assays (HMSA) incorporate an acid dissociation step to separate serum–mAb/ADA complexes and then utilize size-exclusion HPLC to quantify drug and ADA concentrations independently. However, HMSA tests are expensive and require three days to generate results. Concentration and ADA tests for IFX, ADL, vedolizumab, and ustekinumab are available commercially (96,97). An LC/MS/MS method has also been recently developed (98).

The current mAb TDM protocol involves two strategies: either the patient must have blood drawn approximately 1–2 weeks before treatment to allow time for sample shipment and running the assay, or the patient has a sample drawn at the clinic visit, and that data is utilized at the subsequent visit. Taking a sample prior to a clinic visit requires an additional medical appointment. Also, because the TDM measurement is not taken at trough, which is when most publications established for target drug levels, translating the resulting TDM measurement into dose adjustment is more difficult. In the second case, dose adjustments or treatment changes would not be made until the patient’s next dose, generally 2–8 weeks later, depending on the drug.

### Point-of-Care TDM Assays for Biologics

Point-of-care (POC) TDM may be particularly valuable in acute settings such as severe ulcerative colitis. Such patients often clear mAb more rapidly due to drug loss through the gut (protein-losing enteropathy) (99) and dose escalation is a common approach, as discussed previously. POC TDM would provide real-time drug-level information to inform the decision to dose escalate patients to achieve target drug levels, switch therapies, or consider alternative treatment modalities such as surgery.

POC TDM is currently unavailable for mAb therapeutics in the USA. CE-marked tests for IFX are commercially available in Europe (100,101). Both utilize a sandwich immunoassay format. An anti-idiotype antibody is used for capture on the lateral flow membrane and recombinant TNF-α conjugated to gold colloid as the detection reagent.

MAb TDM tests that utilize mimetope peptides as capture or detection reagents in ELISA and lateral flow immunoassay (POC device) are also being developed. Peptides specific for rituximab and alemtuzumab were originally described (102,103), and many others have been developed. These peptides, which are referred to as Veritopes, are selected via phage display against mAb targets (novel or biosimilar) or other proteins, such as receptor fusions. They act as surrogate ligands, binding at the active sites of the antibodies. Veritopes are specific for their target mAb and compatible with serum, plasma, and whole blood. Veritopes offer several benefits over current reagents used in TDM, such as anti-idiotype antibodies and recombinant proteins:

1. Veritope selection is rapid and does not require immunization of animals.
2. Veritopes are easily synthesized and inexpensive to produce.
3. Veritope peptides are easily modified by traditional methods (biotinylation, acetylation, fluorescent labeling).

In contrast, recombinant protein expression is not always feasible and anti-idiotype antibody identification is tedious and not always successful. Large-scale production of recombinant proteins and anti-idiotype antibodies are also costly and stability may be an issue with the former. Unlike cell-based assays, mass spectrometry, and HMSA, Veritopes are amendable to multiple immunoassay formats that do not require highly trained users or expensive equipment. Finally, Veritopes are surrogate ligands that only recognize active (unbound) drug, unlike mass spectrometry, which quantitates all drugs (e.g., active and inactive) in the sample.

### Assays for Anti-drug Antibodies

Laboratory results for ADA are highly dependent on the assay used to measure ADA. Historically, ADA assays are prone to interference by the presence of drug in samples and sensitive assays only detect ADA in the presence of low amounts of drug (104). However, recent technology advancement has overcome the limitation of drug interference experienced by many previous ADA assays (105). Reported results from immunogenicity assays increasingly include the intensity of ADA expressed as semi-quantitative titer values or other units (106).

## CONCLUSIONS

MAbs such as IFX and ADL have proven to be effective in the treatment of auto-immune diseases. However, a significant proportion of patients do not respond to induction treatment or lose response during maintenance, presumably because of inadequate drug exposure. TDM, with the use of dashboard systems, can contribute to adequate dosing by taking into account specific patient factors influencing mAb clearance. Clinical trial data can often illustrate the *E*–*R* relationship for mAbs, although this is more often when the data come from a wide dose range and include data from numerous patients such as phase 3 studies. The *E*–*R* curves may suggest that a higher responder rate is associated with higher exposure, (e.g., steady-state trough concentration), and steeper *E*–*R* curves may suggest greater potential benefit with dose intensification. However, whether intensified dosing regimens will lead to greater clinical efficacy with acceptable safety profiles remains to be experimentally demonstrated because extrapolation of efficacy and safety is challenging. Moreover, understanding patient factors that would drive the treatment response remains an area for future research.

In summary, TDM is a growing part of the precision medicine approach. Evidence supporting correlation of interpatient PK variation with treatment outcomes is extensive and growing. As combination therapy use increases in the treatment of many diseases, PK variability and factors influencing individual patient exposure are generally different between drugs, and therefore, exposure to different drugs in a combination setting may ultimately need to be addressed. While not currently available for all biologics, POC TDM is warranted in certain circumstances and enables personalized, precision dosing. Most current methods for TDM are not amenable for rapid POC testing, but alternative technologies are being developed to make POC TDM reliable and practical for patients treated with biologic therapies. An unmet clinical need exists to mitigate the risks of interpatient PK variability among patients treated with mAbs.

## Figures and Tables

**Table I.** List of Dose Regimens of Monoclonal Antibody Drugs Approved for the Treatment of Crohn’s Disease and Ulcerative Colitis

Drug name (approval year)	Induction treatment Indication, route of administration, dose regimen	Maintenance treatment Indication, route of administration, dose regimen
Infliximab (1998)	(CD) IV, 5 mg/kg @ W0, 2, 6	(CD) IV, 5 mg/kg Q8W (option to increase to 10 mg/kg)
	(UC) IV, 5 mg/kg @ W0, 2, 6	(UC) IV, 5 mg/kg Q8W
Certolizumab pegol (2008)	(CD) SC, 400 mg @ W0, 2, 4	(CD) SC, 400 mg Q4W
Natalizumab (2008)	(CD) 300 mg Q4W; discontinue in patients that have not experienced therapeutic benefit by 12 weeks of induction therapy.	
Adalimumab (2007, 2012)	(CD, UC) SC, 160 mg @ W0, 80 mg @ W2, 40 mg @ W4	(CD, UC) SC, 40 mg Q2W
		(UC) continue only if clinical remission by W8)
Golimumab (2013)	(UC) SC, 200 mg @ W0, 100 mg @ W2	(UC) SC, 100 mg Q4W
Vedolizumab (2014)	(CD, UC) IV, 300 mg @ W0, 2, 6	(CD, UC) IV, 300 mg Q8W (discontinue if no benefit by W14)
Ustekinumab (2016)	(CD) IV, dosing by weight (3 tiers)	(CD) SC, 90 mg Q8W

*W* week, *Q2W* once every 2 weeks, *Q4W* once every 4 weeks, *Q8W* once every 8 weeks, *CD* Crohn’s disease, *UC* ulcerative colitis

**Table II.** Potential Reasons for Treatment Failure Grouped by the Measurements of ADA and Drug Concentration

Drug concentration	ADA− (or low ADA titer)	ADA+ (or high ADA titer)
High (or therapeutic level)	May be related to mechanism of action of drug^a^	May be related to mechanism of action of drug^a^
Low (or subtherapeutic level)	May be suboptimal dose, non-immune-mediated PK issue	May be immune-mediated PK issue

a Such as patients not responsive or with low sensitivity to the mechanism of action of the drug
